# Artificial Abortion

**Published:** 1880-08-20

**Authors:** M. H. Jordan


### A CASE OF ARTIFICIAL ABORTION FOR RELIEF OF UNCONTROLLABLE NAUSEA AND VOMITING, WITH REMARKS.

BY M. H. JORDAN, M.D.

Mrs. C. H. T., aged 24 years, with a good family history, enjoyed excellent health until three or four years ago, when she received a fall by a chair being jerked from under her. Since which time she has suffered from a displacement of the uterus, complicated with a cystitis and reflex irritation about the rectum.

This lady was treated for these troubles several years ago by Dr. E. W. Jenks, of Chicago, but removed to this state before a complete cure was established. The subject of this paper, although suffering from uterine, cystic and rectal troubles, married about eight months ago, and in three or four months became pregnant.

On June 23, 1879, I was telegraphed to visit Mrs. S., at her home in Shelby county, Alabama, and learned that she had been confined to her bed for five weeks, with a distressing and uncontrollable nausea and vomiting. She was much emaciated, very nervous, with a quick pulse, some fever, and retained comparatively nothing on the stomach, and had but little quiet and undisturbed sleep either day or night. There was great soreness over the stomach and abdomen, and the act of vomiting was frequent and distressing.

I tried all of the remedies that are usually given in such cases with negative results; and in order to secure a night's rest I administered one-fourth of a grain of morphia hypodermically, but it seemed to increase the nausea and vomiting, and produced such alarming nervous symptoms that I was deterred from again attempting its use. Among a large number of other drugs, I gave large doses of the bromide of potassium and hydrate of chloral by enema, with no other result than to increase the local irritation already existing in the rectum, and causing additional pain and constitutional disturbance.

Being baffled and disappointed thus far in everything, I concluded to try a remedy that I believe was first suggested by Dr. J. Marion Sims —that of applying nitrate of silver to the uterus. Accordingly, a Sims's speculum was introduced, and a thorough application made to a reddened and inflamed os and cervix uteri; but this remedy produced no other effect than a slight discharge from the vagina for several days.

Seeing that the patient's condition was growing more critical, and her strength failing day by day, I concluded that the time had certainly arrived to attempt an abortion. So accordingly I passed a sound to the fundus of the uterus. I felt sure that this would end my troubles with this case, for I fully expected to receive intelligence in a few days, either from the husband or attending physician, that the lady had miscarried, but instead was summoned to visit her again, as the treatment had produced no effect.

At this visit I found the patient still more exhausted from loss of sleep and rest, and retained scarcely any food or drink, and it was evident that without relief she certainly could not long survive; so I advised her husband to convey her to the train (on a mattress), and thence to Birmingham, where I could see her often, and give the necessary attention.

On the following morning I called Drs. J. W. Sears and W. P. Taylor in consultation, and we found the patient in the following condition, viz.: Distressing nausea and vomiting, a feeble pulse (from one hundred and twenty to one hundred and forty beats per minute), a hot, dry skin, a dry coated tongue, parched lips, and presenting the appearance very much of a patient in the fourth week of typhoid fever. Drs. Sears and Taylor agreed with me that abortion was certainly justifiable, and should be brought on as soon as possible; consequently I again passed the sound into the uterus, packed the vagina with a cotton tampon, and gave large doses of ergot hypodermically, but even this medication and local irritation proved insufficient to arouse the uterus to contraction.

As time was a great consideration in this case, and fearing the evils of delay, on the next day I passed a small sea-tangle tent well into the uterus, and tamponed the vagina as before, so as to hold the tent *in situ*, and let it remain fifteen hours; but this produced no other result than slight dilatation of the os uteri.

Seeing that unless relieved this lady could not live many days, as she had become so weak that she could not turn herself in bed, we resolved upon active interference to empty the uterus of its contents, and lessen the tension on the uterine fibers. In obedience to this resolution, I introduced a large sponge tent into the uterus beyond the os internum uteri, and secured it in place as before with a cotton tampon.

At the expiration of six hours I introduced a Sims's speculum, and after removing the cotton and sponge tent, which had done its work well and fully dilated the cervical canal, I hooked a uterine tenaculum into the anterior lip of the cervix, and slowly but steadily drew it down near the vulva, and within easy reach of my fingers. I then withdrew the speculum (the tenaculum remaining), and had the patient turned on her back, her hips drawn well over the edge of the bed, her legs flexed, and thighs held at right angles with the body, so as to secure the greatest degree of relaxation possible to the perineum and abdominal muscles, and introducing two fingers into the cavity of the uterus removed a fœtus (a miniature baby) at about the fourteenth week of utero-gestation. Then with my index finger I made the complete circuit of the uterine cavity, and removed the little placenta; and wishing to sustain all the strength the patient had, I injected about a pint of hot water into the uterus to secure a good contraction and prevent hemorrhage.

Dr. Sears kindly gave the patient a little chloroform during the operation, and she rallied well and experienced but little shock. In several hours I called and found the patient suffering considerably from after-pains; but after removing a small clot from the cervical canal, I washed out the vagina with tepid carbolized water, and gave fifteen minims of the fluid extract of hyoscyamus hypodermically (as she could not take morphia), which secured her a good night's rest—the only one that she had received for seven or eight weary weeks.

After the operation it was indeed wonderful what a transformation there was in this patient's condition; for she never became sick at the stomach nor vomited a single time, but immediately began to retain milk and absorb all that was given her. I used no other treatment save an antiseptic vaginal wash; and she steadily improved, her fever subsided, her tongue became moist and began to clean off, appetite returned, and at the end of two weeks was able to be carried home with her mother to Chattanooga, Tennessee.

*Remarks.*—In any case of abortion where the decidua has not been expelled, the obstetrician has not fully discharged his duty until he has by bold and prompt measures removed it by manual extraction. This operation does not require any great amount of technical skill, and its immediate results are in the highest degree satisfactory. It can be accomplished by placing the patient crosswise in bed, her hips near the edge, her legs flexed and thighs held at right angles to the body; and with the left hand over the symphysis pubis press the uterus well down into the cavity of the pelvis, and pass the index finger of the right hand through the cervix, and by making the complete circuit of the uterine cavity the decidua can be removed without much difficulty.

In many ordinary cases of abortion the placenta is left *in utero*, to be thrown off by nature as best she may; and doubtless the large number of helpless, broken-down women, with long histories of repeated hemorrhages, fetid discharges, and local inflammations—either a subinvolution, or uterine or cervical catarrh—who present themseves almost daily to their family physician, or finally seek relief of some specialist in the larger cities, are in as many instances due to bad management in abortion as in labor at full term.

I do not believe that any woman is safe in abortion, nor should the obstetrician leave his patient until the little placenta has been removed in the manner just detailed; for in my limited gynecological practice I have seen cases of serious uterine trouble caused by a small tuft of the placenta left *in utero* after an abortion, and which was relieved after its removal by Thomas's curette, combined with constitutional treatment. For the extraction of the placenta the index finger is preferable to the ovum forceps, for it is safer, simpler, and there is no danger of producing local injury to the endometrium.

In conclusion, the following summary of views are respectfully offered in connection with this case :

1. We could hardly conceive of a more unfavorable case for operative interference than the one just detailed, and the result tends to justify so bold a procedure in such cases.

2. If this lady had not have aborted she certainly would have died.

3. The vital powers were so completely overwhelmed by the constitutional disturbance of nausea and vomiting, that nature either refused or was unable to arouse sufficient uterine energy to expel its contents.

4. In all cases where nature fails to bring about abortion, and the patient's life is in jeopardy, if the obstetrician does not empty the uterus of its contents by a bold and prompt procedure, he has not given his patient the benefit of all the resources of his vast art.

I hereby return thanks to Drs. Taylor and Sears, for valuable counsel and assistance in the management of this case.

